# Supplementary material for: The Associations between Evacuation Movements and Children’s Physiological Demands Analyzed via Wearable-Based Sensors
Source: Sensors (Basel). 2022 Oct 22;22(21):8094. doi: 10.3390/s22218094 (PMC9658188; doi:10.3390/s22218094)
Supplement: Supplementary file 1 [file sensors-22-08094-s001.zip › sensors-1908584-supplementary.pdf]

Supplementary material

The associations between evacuation movements and children's physiological demands analyzed via wearable-based sensors

Bo Zhang [1], Xiaoyu Gao [1], Jiaxu Zhou [2,*] and Xiaohu Jia [1,3,*]

[1] Architecture College, Inner Mongolia University of Technology (IMUT), Hohhot 010051, China
[2] UCL Institute for Environmental Design and Engineering, The Bartlett, University College London (UCL), London WC1H 0NN, UK
[3] Inner Mongolia Key Laboratory of Green Building, Architecture College, Inner Mongolia University of Technology (IMUT), Hohhot 010051, China
* Correspondence: jiaxuzhou@yeah.net (J.Z.); jiaxiaohu@imut.edu.cn (X.J.)

The raw data of children's evacuation speed in knee and hand crawling (KHC) condition.

In this study, the evacuation distance was 22.0 m, and the evacuation velocity was calculated by Formula: $V = S/\Delta T$, where V (m/s) stands for the velocity, S (m) stands for distance, and $\Delta T(s)$ stands for the time.

Table S1. The data for boy's velocity in KHC

Number	Time	Velocity
1	33.191	0.663
2	19.236	1.144
3	34.510	0.637
4	24.068	0.914
5	26.651	0.826
6	35.069	0.627
7	29.965	0.734
8	49.546	0.444
9	57.357	0.384
10	43.479	0.506
11	43.137	0.510
12	36.667	0.600
13	24.719	0.890

KHC—knee and hand crawling.

Table S2. The data for girl's velocity in KHC

Number	Time	Velocity
1	32.812	0.670
2	46.002	0.478
3	53.275	0.413
4	50.594	0.435
5	46.153	0.477
6	51.377	0.428
7	55.279	0.398
8	53.120	0.414
9	48.831	0.451
10	34.213	0.643
11	50.946	0.432
12	50.000	0.440
13	48.889	0.450
14	48.915	0.450

KHC—knee and hand crawling.

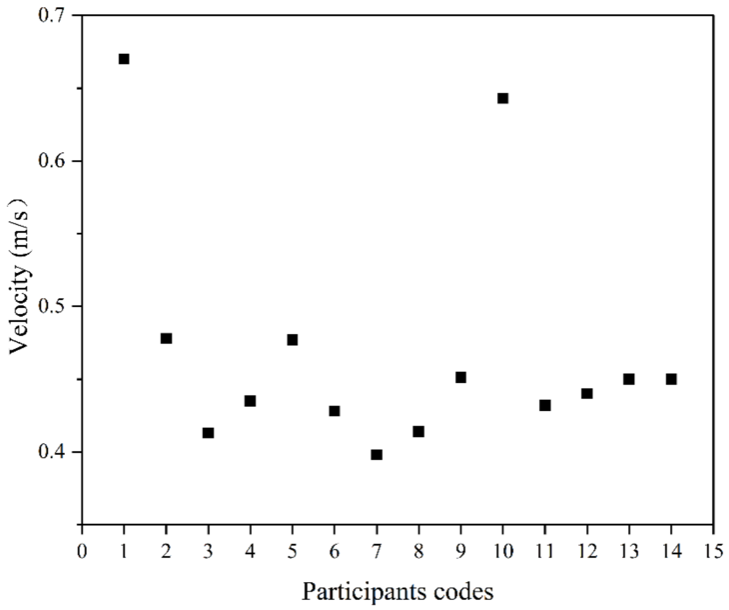

Figure S1. The crawling velocities for girls.

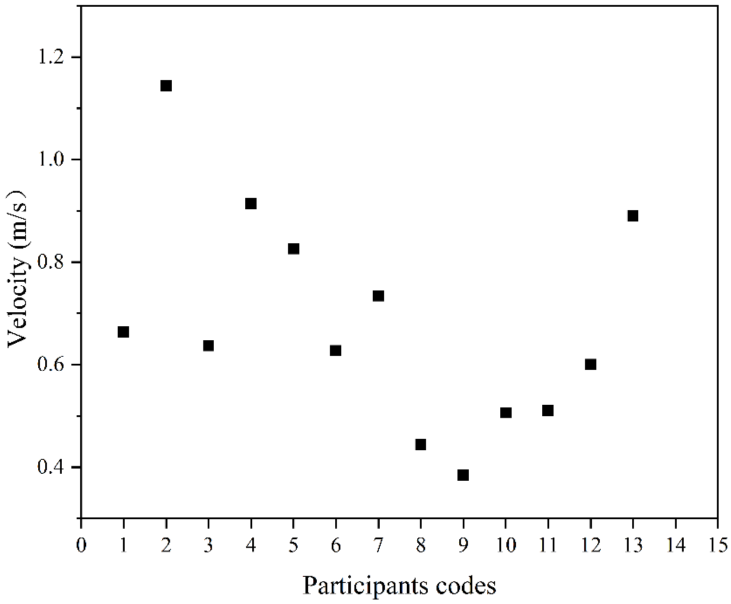

Figure S2. The crawling velocities for boys.

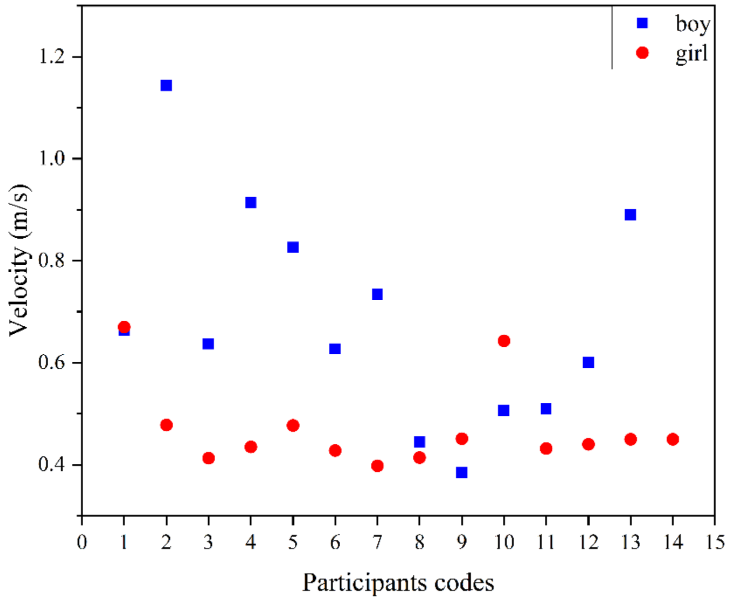

Figure S3. The scatterplot of velocity for subjects.
